# Dual-emissive, oxygen-sensing boron nanoparticles quantify oxygen consumption rate in breast cancer cells

**DOI:** 10.1117/1.JBO.25.11.116504

**Published:** 2020-11-23

**Authors:** Ashlyn G. Rickard, Meng Zhuang, Christopher A. DeRosa, Xiaojie Zhang, Mark W. Dewhirst, Cassandra L. Fraser, Gregory M. Palmer

**Affiliations:** a Duke University, Department of Radiation Oncology, Duke University Medical Center, Durham, North Carolina, United States; b University of Virginia, Department of Chemistry, Charlottesville, Virginia, United States

**Keywords:** boron nanoparticles, oxygen imaging, oxygen consumption rate, breast cancer, dual-emission microscopy, irradiation

## Abstract

**Significance:** Decreasing the oxygen consumption rate (OCR) of tumor cells is a powerful method for ameliorating tumor hypoxia. However, quantifying the change in OCR is challenging in complex experimental systems.

**Aim:** We present a method for quantifying the OCR of two tumor cell lines using oxygen-sensitive dual-emissive boron nanoparticles (BNPs). We hypothesize that our BNP results are equivalent to the standard Seahorse assay.

**Approach:** We quantified the spectral emissions of the BNP and accounted for external oxygen diffusion to quantify OCR over 24 h. The BNP-computed OCR of two breast cancer cell lines, E0771 and 4T07, were compared with their respective Seahorse assays. Both cell lines were also irradiated to quantify radiation-induced changes in the OCR.

**Results:** Using a Bland–Altman analysis, our BNPs OCR was equivalent to the standard Seahorse assay. Moreover, in an additional experiment in which we irradiated the cells at their 50% survival fraction, the BNPs were sensitive enough to quantify 24% reduction in OCR after irradiation.

**Conclusions:** Our results conclude that the BNPs are a viable alternative to the Seahorse assay for quantifying the OCR in cells. The Bland–Altman analysis showed that these two methods result in equivalent OCR measurements. Future studies will extend the OCR measurements to complex systems including 3D cultures and *in vivo* models, in which OCR measurements cannot currently be made.

## 1 Introduction

The oxygen consumption rate (OCR) provides quantitative information regarding cellular metabolism and the utilization of oxygen. Oxygen is required for normal tissue function and is directly involved in the production of adenosine triphosphate (ATP) through its role in the electron transport chain.^1^ In cancer, a well-oxygenated tumor is more amenable to a therapeutic response, while hypoxic tumors (tumor tissue ${\mathrm{pO}}_{2}<10\;\mathrm{torr}$) are associated with poor therapeutic outcomes. Hypoxia develops as a result of an imbalance of oxygen supply and demand, and there is no standard method in current clinical practice to decrease hypoxia in cancer though there are a myriad of treatments (i.e., anti-angiogenetic agents) to reduce tumor hypoxia. However, one possibility for ameliorating the problem of hypoxia is to decrease the OCR of tumor cells;^2^^–^^5^ Secomb et al,^6^ using a mathematical model of tumor blood vessels, predicted that a decrease of 30% in the OCR of tumor cells would abolish hypoxia; compared with increasing oxygen delivery, decreasing the OCR could be 30× more efficient. To use this therapy, a reliable, accurate method for measuring the OCR both *in vitro* and *in vivo* is necessary. Measuring OCR is complex; *in vivo* characterization of whole-tumor OCRs includes multiple factors that bias the data: there are strong dependences on the number of cells consuming oxygen, the type of cells,^7^ the presence of any mitochondrial mutations,^8^^–^^10^ the relative tumor vascularization,^10^^–^^12^ and the differentiated state of the cells (non-differentiated cells are more glycolytic).^13^ *In vivo* measurements of tumor OCRs are useful in characterizing the entire tumor microenvironment, but it is nearly impossible to remove the effects of perfusion and oxygen consumption of stromal and immune cells to isolate and measure the tumor-cell OCR alone.^14^^–^^16^

*In vitro* oxygen consumption measurements are standardly performed using the Seahorse assay, which removes these *in vivo* variables. The Seahorse assay is used to measure the mitochondrial respiration rate by measuring the OCR before and after the addition of various inhibitors.^17^ The major defect in this assay is its lack of accuracy^18^—the system is not airtight as it uses oxygen-permeable materials, and the diffusion of external oxygen into the cell chamber can affect measurements.^19^^,^^20^ Compartmental models have decreased the impact of oxygen diffusion to some extent.^21^ Moreover, due to the inherently large number of variables that affect OCR [plating conditions, days of growth, medium composition (both growth and assay), and concentrations of mitochondrial effectors], inter-laboratory differences have been reported, and care must be taken in the manner of presenting the data.^22^ In addition, there is no spatial information in Seahorse data, whereas there is potential for fluorescence-based microscopy OCR measurements to include this information.

In oncology, it is well-documented that tumor cells exhibit increased glycolysis and decreased mitochondrial activity—both drivers for changes in oxygen consumption.^10^ Importantly, several studies have extended these concepts to find that tumor growth can be regulated through the modulation of the OCR.^10^^,^^16^^,^^23^ This modulation can evoke various levels of response to standard therapies; however, radiation is of particular interest because of the extreme resistance of hypoxic tumors to radiotherapy. Several studies have successfully modulated mitochondrial activity to act as a radiosensitizer.^24^^–^^26^ Harnessing this knowledge for therapeutic uses requires consistent, repeatable, precise measurements of the OCR and understanding of how modulating it affects radiation therapy outcomes.

Ratiometric methods for measuring OCR *in vitro* have become increasingly popular due to increased resolution and real-time analysis.^18^ This is done through various mechanisms, usually with indirect oxygen measurements made by redox-sensitive probes or by quantifying the amount of reactive-oxygen-species.^27^^–^^30^ Herein, we present a novel ratiometric method for quantifying *in vitro* OCRs using dual-emissive, oxygen-sensing boron nanoparticles (BNPs) that directly report oxygen concentration. The BNPs are fabricated from difluoroboron β-diketonate (${\mathrm{BF}}_{2}\mathrm{bdk}$)-based dye-polymer conjugates. Due to high quantum yields, large extinction coefficients, and tunable emission colors, ${\mathrm{BF}}_{2}\mathrm{bdk}$ dyes have served as versatile tools for imaging and optics reagents.^31^^–^^35^ When the dyes are confined in a rigid polymer matrix, such as poly(lactic acid) (PLA), both fluorescence and room-temperature phosphorescence are present: the fluorescence remains unaffected by changes in oxygen concentration, but the phosphorescence is sensitive to changes in environmental oxygen via collisional quenching.^36^^,^^37^ The fluorescent and phosphorescent emissions are independent, with lifetimes at 462 and 569 nm under $N_{2(g)}$ to be 0.47 ns, and 1.9 ms, respectively. This is strong evidence that the short-lived, blue emission is fluorescence from the singlet excited-state and the long-lived, yellow emission is phosphorescence originating from the triplet-excited state. Furthermore, the yellow emission is quenched in the presence of oxygen, whereas the blue emission is unchanged according to total emission spectroscopy. While there is some spectral overlap between the two features, this is minimal.^38^ This unique feature makes the ${\mathrm{BF}}_{2}\mathrm{bdk}$ materials useful probes in ratiometric imaging. By taking the ratio of fluorescence to phosphorescence, one can determine the concentration of oxygen in the sample while controlling for effects of particle heterogeneity, illumination intensity, and collection efficiency. In the dye-polymer conjugates, the ${\mathrm{BF}}_{2}\mathrm{bdk}$ chemical structures are related to dye properties (e.g., emission color and oxygen detection range). For example, an iodide-substituted difluoroboron dibenzoymethane poly-L-lactide or PLLA (PLA) material $\left[{\mathrm{BF}}_{2}\mathrm{dbm}(I)\mathrm{PLA}\right]$^32^ can sense the oxygen level from 0% to 21%, while a dinapthoylmethane dye-polymer $\left[{\mathrm{BF}}_{2}\mathrm{dnm}(I)\mathrm{PLA}\right]$^39^ is suitable for hypoxia (0% to 5%). The majority of physiologically based applications will occur in the 1% to 11% $O_{2}$ range.^40^ We have previously demonstrated the use of these two types of BNPs in *in vivo* murine dorsal window chambers to measure intracellular ${\mathrm{pO}}_{2}$ and tumor hypoxia in the microenvironment.^32^^,^^39^ Recently, an iodide-substituted napthyl-phenyl derivative dye $\left[{\mathrm{BF}}_{2}\mathrm{nbm}(I)\mathrm{PLA}\right]$ showed excellent full range oxygen detection (0% to 100%) and was applied to monitor wound healing.^38^ In addition to using hydrophobic PLA as a rigid matrix, hydrophilic polymers such as poly(ethylene glycol)(PEG) are also used to create nanoparticles with improved aqueous stability. PEGylated nanoparticles also have an advantage in passive tumor targeting in which they are taken up by cells via the EPR effect. We have reported a strategy to assemble stereocomplexed PEGylated nanoparticles from boron dye-PLLA-PEG and PDLA-PEG with tumor accumulation in a murine model.^37^ In this study, we covalently linked the full range boron dye to PLLA-PEG and applied the established PEGylation method to generate oxygen-sensing BNPs for measuring OCR.^37^

Our method fills an important gap in metabolic measurements because, while standard Seahorse techniques can be expanded from 2D monolayers of cells to *ex vivo* 3D tissues, they rely on inducing changes in oxygen consumption by inhibitors of mitochondrial function. Our proposed method translates from standard 2D cultures to 3D organs. We have previously used BNPs for straightforward $\%O_{2}$ imaging in *in vivo* murine dorsal window chamber models that accommodate the depth limitations of optical wavelengths.^33^^,^^38^ Our objective here is to measure and compare the *in vitro* OCR measured in two breast cancer cell lines, E0771 and 4T07, with the gold-standard Seahorse assay. This is the first step toward our ultimate goal of extending these OCR measurements *in vivo*. To measure the OCR, BNPs are added to cell media (or any other experimental solution) and their phosphorescence and fluorescence intensity is measured in a spectroscopic plate reader. In our study, we also irradiated each cell line to reach 50% survival to determine if the basal OCR is changed. We hypothesize that (1) OCRs of E0771 and 4T07 can be quantified via ratiometric sensing with the BNP, (2) OCRs measured via the plate reader are comparable to the gold-standard Seahorse assay, and (3) OCR is reduced after a dose of radiation that reduces survival by 50%.

## 2 Experimental Methods and Materials

### 2.1 BNP Synthesis Materials and Methods

The lactide monomer (D-lactide) was a generous gift from Corbion Purac©. L-Lactide was purchased from TCI America. Lactide was recrystallized twice from EtOAc and dried in vacuo overnight prior to use. PEG was purchased from Sigma Aldrich (2 000 Da, Đ=∼1.05) and dried via azeotropic distillation in toluene according to a previously described protocol.^41^ The PEG was stored under nitrogen in a glovebox prior to use as a macroinitiator. The polymers PEG-PLLA-OH (GPC: $M_{n}=6\;900$ Đ=1.04, and H1 NMR=7 000) and PEG-PDLA-OH ($M_{n}=12\;500$, Đ=1.08, and H1 NMR=13 800)^36^ and the ester, 6-iodo, 2-methylnaphthoate^42^ were prepared as previously described. H1 NMR spectra are in accord with literature values. Solvents ${\mathrm{CH}}_{2}{\mathrm{Cl}}_{2}$ and THF were dried over 3 Å molecular sieves activated at 300°C, transferred via cannula, and dried a second time over 3 Å molecular sieves activated at 300°C.^43^ The solvents were stored in a dry pot. All other chemicals were reagent grade from Sigma-Aldrich and were used without further purification.

Synthetic Methods H1 NMR spectra (600 MHz) were recorded on a Varian VMRS 600/51 instrument in ${\mathrm{CDCl}}_{3}$ or $D_{6}\text{-}\mathrm{DMSO}$. H1 NMR spectra were referenced to the residual signals for protiochloroform (7.26 ppm), protioDMSO (2.50 ppm), and protioacetone (2.09 ppm). In the H1 NMR assignments, aromatic positions are defined for phenyl (Ph) and naphthyl (Np) positions. Coupling constants are given in hertz. Number average molecular weights ($M_{n}$), weight average molecular weights ($M_{w}$), and polydispersity index (Đ) were determined by gel permeation chromatography (GPC) (THF, 25°C, 1  ml/min) using multiangle laser light scattering (λ=658  nm, 25°C) and refractive index (λ=658  nm, 25°C) detection. A Polymer Laboratories 5  μm mixed-C guard column and two GPC columns along with Wyatt Technology Corp. (Optilab REX interferometric refractometer, miniDawn TREOS laser photometer) and Agilent Technologies instrumentation (series 1260 HPLC) and Wyatt Technology software (ASTRA 6.0) were used for analysis. The incremental refractive index (dn/dc) was determined by a single-injection method assuming 100% mass recovery from the columns. UV–vis spectra were recorded on a Hewlett–Packard 8452A diode-array spectrophotometer.

**Fig. 1:**
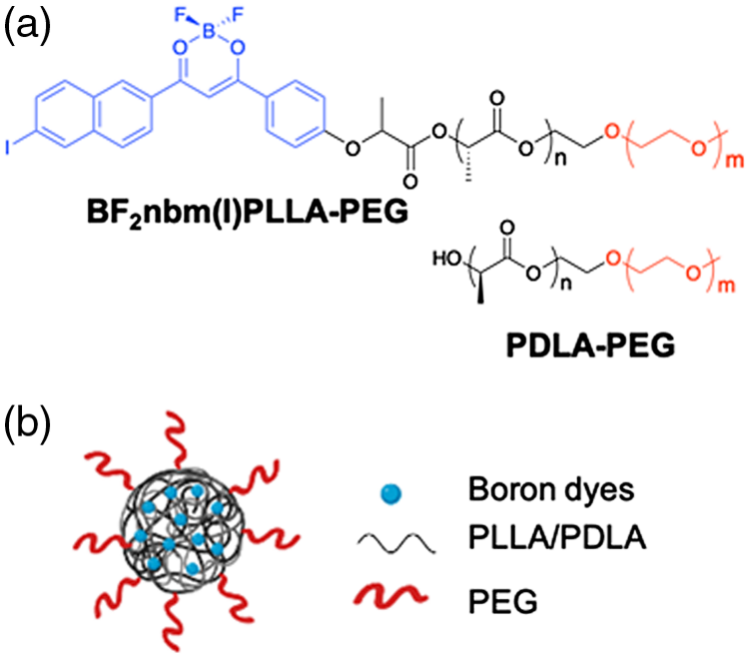
Nanoparticle design and composition of stereocomplex nanoparticles: (a) chemical structures of BF2nbm(I)PLLA-PEG and PDLA-PEG polymers. (b) Schematic illustration of stereocomplex PEGylated nanoparticles.

Figure 1 shows the stereocomplex nanoparticle design and composition.

### 2.2 Polymer Synthesis

*nbm(I)OH*: The iodo-substituted, naphthyl-phenyl ligand was prepared as previously described by Jin et al,^44^ for 3-hydroxy-1-(4-hydroxyphenyl)-3-phenylprop-2-en-1-one, except the aromatic ester 6-iodo, 2-methylnaphthoate was used in place of methyl benzoate to yield a dark brown powder; 178 mg (13%). H1 NMR: (600 MHz, $D_{6}\text{-}\mathrm{DMSO}$) δ17.38 (s, 1H, enol-O*H*), 10.47 (s, broad, 1H, phenol-OH), 8.75 (s, 1H, 1-NpH), 8.48 (s, 1H, 5-NpH) 8.18 (d, J=12, 3-NpH), 8.08 (d, J=12, 2H, 2, 6-PhH), 7.98 (d, J=12, 1H, 4-NpH) 7.85 (s, broad, 2H, 7, 8-NpH), 7.34 (s, 1H, COCHCO), 6.90 (d, J=12, 2H, 3, 5-PhH) (Fig. S2). HRMS [electrospray ionization (ESI), time-of-flight (TOF)] m/z calcd for $C_{19}H_{12}O_{3}I$: 414.9831 ${\left[M-H\right]}^{+}$; found 414.9825.

*BF_2_nbm(I)OH*: The boron dye coupler was prepared as previously described^37^ but with nbm(I)OH to yield a yellow powder after recrystallization from acetone/hexanes; 45 mg (35%). 1H NMR: (600 MHz, $D_{6}\text{-}\mathrm{DMSO}$) δ11.22 (s, broad, 1H, phenol-OH), 8.99 (s, 1H, 1-NpH), 8.55 (s, 1H, 5-NpH), 8.33 (m, 3H, 2, 6-Ph*H*, 3-NpH), 8.07 (d, J=6, 1H, 4-NpH), 7.95 (m, 2H, 7, 8-NpH), 7.87 (s, 1H, COCHCO), 7.00 (d, J=6, 2H, 3, 5-PhH) (Fig. S3). HRMS (ESI, TOF) m/z calcd for $C_{19}H_{11}{\mathrm{BO}}_{3}F_{2}I$: 462.9814 ${\left[M-H\right]}^{+}$; found 462.9814.

*BF_2_nbm(I)PLLA-PEG*: The dye-coupled polymer was prepared as previously described by Kerr et al,^37^ except the dye, ${\mathrm{BF}}_{2}\mathrm{nbm}(I)\mathrm{OH}$, was used in placed of ${\mathrm{BF}}_{2}\mathrm{dbmOH}$. A lower MW mPEG-PLLA-OH block copolymer (PEG=2 000  Da and PLLA=5 000  Da) was also used in this reaction, prepared as previously described.^2^ The product was obtained as a yellow powder; 513 mg (76%). $M_{n}$ (GPC/MALS)=7 600 (dn/dc=0.056), Đ=1.05 (H1 NMR)=7300; H1 NMR: (600 MHz, ${\mathrm{CDCl}}_{3}$) δ8.69 (s, 1H, 1-NpH), 8.32 (s, 1H, 5-NpH), 8.17 (d, J=6, 2H, 2, 6-PhH), 8.09 (d, J=12, 1H, 3-NpH), 7.85 (m, 2H, 7, 8-NpH), 7.72 (d, J=12, 1H, 4-NpH), 7.21 (s, 1H, COCHCO), 7.06 (d, J=6, 2H, 3, 5-PhH), 5.17 (q, J=6, 66H, PLLA-H), 3.62 (s, broad, 179H, $\mathrm{PEG}\text{-}{\mathrm{OCH}}_{2}{\mathrm{CH}}_{2}$-), 3.36 (s, 3H, $\mathrm{PEG}\text{-}{\mathrm{OCH}}_{3}$), 1.55 (m, broad, 217H, $\mathrm{PLLA}\text{-}{\mathrm{CH}}_{3}$) (Fig. S4).

### 2.3 Nanoparticle Fabrication

Stereocomplex nanoparticles, as shown in Fig. 1, were fabricated as previously described.^37^ In brief, oxygen-sensing polymer (${\mathrm{BF}}_{2}\mathrm{nbm}(I)\mathrm{PLLA}\text{-}\mathrm{PEG}$) (45 mg) and mPEG-PDLA (45 mg) were dissolved in DMF (9 ml). The solution was briefly heated to facilitate dissolution of the polymers. With a syringe pump, the DMF solution was added to deionized (DI) water (81 ml) at a constant rate of 1  ml/min. The solution was filtered through Whatman filter paper, and DMF was removed by dialysis (Select/Por; 12 to 14 kDa molecular weight cutoff; 2-l beaker filled with DI water; water changed every 2 h for 6 h, then allowed to stand overnight). The nanoparticle solution was then passed through a Whatman 200-nm Anotop filter. Nanoparticle sizes and polydispersities were analyzed via dynamic light scattering (DLS, Wyatt, DynaPro Plate Reader II), and results were consistent with previous reports (Fig. S5).^25^

### 2.4 Cell Culture

The murine breast cancer cell line E0771 (CH3 Biosystems, Amherst, New York) was cultured in RPMI 1×1640 with 300  mg/L of L-glutamine (Gibco Gaithersburg, Maryland). 4T07 cells were acquired from American Type Culture Collection and cultured in DMEM with 4.5  g/L of D-glucose, 584  mg/L of L-glutamine, and 110  mg/L sodium pyruvate (Gibco Gaithersburg, Maryland). Both cell media were supplemented with 10% v/v heat-inactivated fetal bovine serum (HI-FBS) (Gibco Gaithersburg, Maryland) and 1% v/v anti-mycotic/antibiotic (A/A) (Gibco, Gaithersburg, Maryland). Cells were maintained in a humidified 37°C incubator with 5% ${\mathrm{CO}}_{2}$. Prior to the experiment, two black, 96-well plates with clear bottoms (Greiner, Bio-One, Kremsmünster, Austria) per cell line were plated with $4\times 10^{4}\text{ cells}/\text{well}$ and incubated for 24 h. This resulted in near 100% confluence for each cell line at the time of the study.

### 2.5 Boron Nanoparticle Calibration

**Fig. 2:**
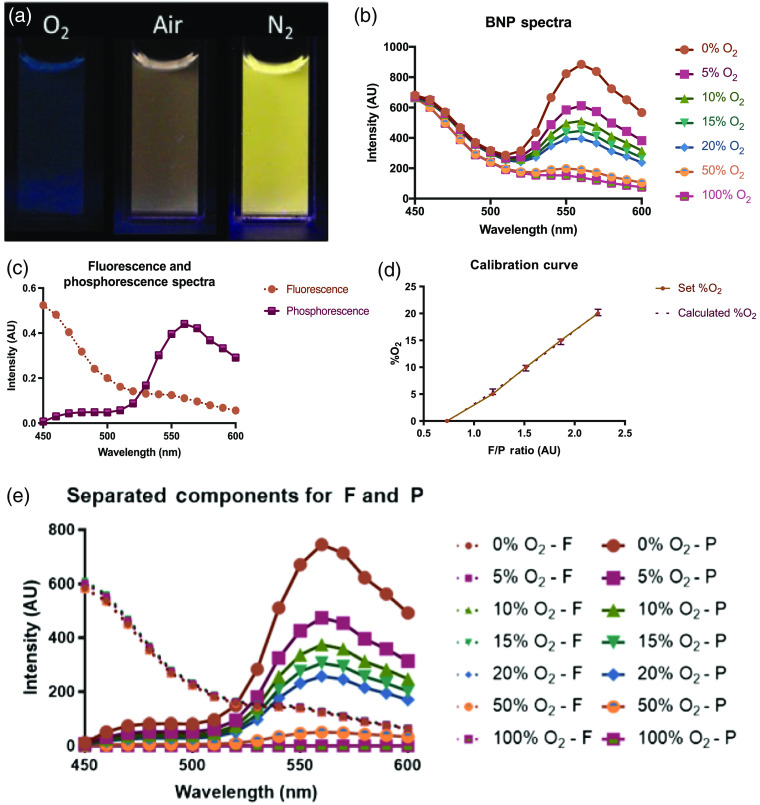
Procedure for BNP Calibration. (a) BNPs in the presence of 100% $O_{2}$ (blue), room air (brown), and 100% $N_{2}$ (yellow) show the color difference as the emission changes in various environments. (b) Raw BNP spectra in the presence of 0%, 5%, 10%, 15%, 20%, 50%, and 100% $O_{2}$. The fluorescence and the phosphorescence are independent; the fluorescence remains steady at 450 nm, and the phosphorescence decreases with increasing oxygen percentage around 560 nm. (c) By decomposing the full BNP spectra (at 5% $O_{2}$) into its separate fluorescent and phosphorescent components that make up the entire signal, the contamination of the independent spectra can be taken into account. (d) The calibration curve shows that the F/P (fluorescence to phosphorescence) ratio against oxygen percentages is quadratic over the entire curve (0% to 100% $O_{2}$). Because our sample will be in the 0% to 21% oxygen percentage range and the BNPs are most sensitive at low oxygenations, our fit is shown from 0% to ∼20% oxygen with standard error bars representing the expected error estimate-based predictions, representing the range of possible $O_{2}$ values, around the fit. (e) The fully decomposed fluorescent and phosphorescent spectra are shown. The solid lines represent fluorescence (F) and the dotted lines represent phosphorescence (P).

To quantify oxygen concentrations in experimental conditions, BNPs were calibrated with a spectrometer (SpectraMax M5, Molecular Devices, San Jose, California). A solution of 1:5 v/v BNP:deionized water was placed in a clear cuvette, and spectra were measured for 0% $O_{2}$, 5% $O_{2}$, 10% $O_{2}$, 15% $O_{2}$, 20% $O_{2}$, 50% $O_{2}$, and 100% $O_{2}$. Oxygen gas was mixed with nitrogen gas to make these defined oxygen concentrations. These gases were bubbled into test solutions for 5 min prior to data acquisition. The top of the cuvette was covered with two layers of Parafilm (American National Can, Menasha, Wisconsin) to minimize oxygen diffusion into the cuvette from the open end. A GFC mass flow controller (Aalborg, Orangeburg, New York) with an oxygen sensor (${\mathrm{MaxO}}_{2}$, Ceramatec Inc., Salt Lake City, Utah) in series with the gas line was used to change the molecular oxygen and nitrogen mixture and to ensure accuracy, respectively. The solution was calibrated at 37°C. The solution temperature was measured with a thermocouple (Model HH23, OMEGA Engineering, Inc., Norwalk, CT). The excitation wavelength was 405 nm. The emission spectrum (450 nm to 600 nm) was measured at 10 nm-step increments [Fig. 2(B)]. This spectrometer system was also used for data acquisition during experiments.

In MATLAB (v R2018b, Mathworks, Natick, Massachusetts), spectrometer data were used for a parallel factor analysis (parafac.m function in N-Way Toolbox^45^) that computes two non-negative intensity components that contribute to each wavelength measurement. These two components are the fluorescence and phosphorescence and are most clearly seen in Fig. 2(C) at 0% $O_{2}$. The component spectra for all oxygen percentages are shown in Fig. 2(E). By breaking down the spectral components of each wavelength and each percent $O_{2}$, the proceeding fluorescent to phosphorescent (F/P) ratio was computed more accurately by determining the exact percentage that each component contributes to the total signal. By repeating this spectral decomposition for each known oxygen concentration, a calibration curve and resulting quadratic fit [Eq. (1), Fig. 2(d)] were determined. This calibration curve was used for the remaining experiments to calculate the percent $O_{2}$ from the measured F/P ratio [Fig. 2(c)]. It was found that a quadratic fit the data well ($R^{2}=0.9985$) and the expected standard error of predictions was <0.6; thus we can use the straightforward relationship shown below:

$$\text{Percent Oxygen}=1.12{\left(\frac{F}{P}\right)}^{2}+10.23(\frac{F}{P})-8.25.$$ (1)

### 2.6 Seahorse Assay

We used previously published work examining oxygen consumption by 4T07 versus E0771 cells via the Seahorse metabolic assay as shown in Ref. 17. In that study, the XF24 Analyzer from Seahorse Biosciences was used to measure the bioenergic profile of 4T07 and E0771 mammary carcinoma cell lines, which were plated in culture to confluency. Real-time measurements of OCR, extracellular acidification rate, and proton production rate were obtained. These mitochondrial function parameters were determined in the presence of mitochondrial uncoupling agents: oligomycin, FCCP (trifluoromethoxy carbonylcyanide phenylhydrazone, an uncoupler that disrupts ATP by moving protons across cell membranes), and antimycin. Of note, these agents were injected sequentially through ports in the XF Assay cartridges to final concentrations of 1  μg/ml, 1  μM, and 10  μM, respectively. The addition of oligomycin, an ATPase inhibitor, shows OCR associated with ATP production. The maximal respiratory rate as reflected by oxygen consumption is determined by the FCCP injection, which transfers $H^{+}$ across the cell membrane prior to use for oxidative phosphorylation. Finally, the addition of rotenone, which inhibits complex I, halts mitochondrial respiration. The study allowed for the determination of baseline mitochondrial activity, OCR from ATP production, and maximal respiration.

### 2.7 Experimental Setup for BNP OCR Assessment

**Fig. 3:**
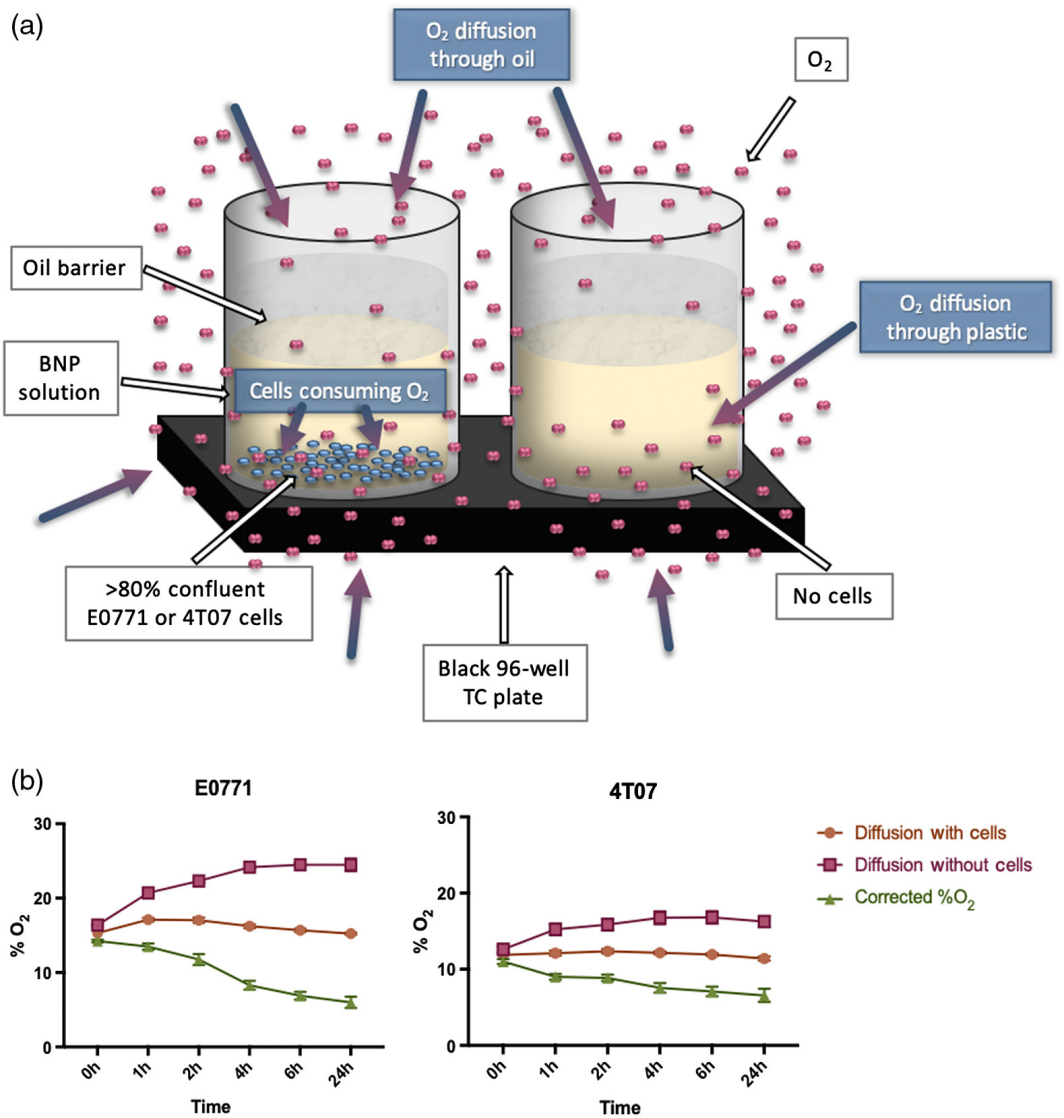
Diffusion and corrected oxygen data for E0771 and 4T07: (a) Despite the oil barrier that was placed on top of the experimental solution (both with cells or without), there is significant external oxygen diffusing into the wells. For the two cells lines E0771 and 4T07, we corrected for the diffusion by measuring the $\%O_{2}$ with and without cells. (b) The difference between these measurements results in the actual $\%O_{2}$ in the solution with cells. This is the first step in calculating the OCR.

The 96-well black plates were setup with twelve wells for each experimental condition, six wells with cells and six wells without cells as shown in Table S1 (Supplemental Material). The volume of BNP solutions covering the wells was 50  μl. The volume of a mineral oil barrier that slowed the external oxygen diffusion into the system during imaging was 100  μl (Fig. 3). Each solution consisted of 20% v/v BNPs. The controls for the experiment were four wells of BNPs, BNPs with mineral oil, and mineral oil alone. The experimental conditions include the following solutions: BNPs and cell media with cells; BNPs and cell media without cells; and BNPs, cell media, and 20 mM of sodium dithionite without cells. The latter, an oxygen scavenger, acted as a positive control. All experimental cell media were supplemented with 10% v/v HI-FBS, 1% v/v A/A and were phenol-red free media (either RPMI or DMEM) (Gibco Gaithersburg, MD).

### 2.8 Irradiation of Cells

An x-ray irradiator (X-RAD 320, Precision X-Ray, North Branford, CT) was used for the irradiation portion of each study. During transport, cells were covered and remained at approximately 37°C via a paraffin wax pad. Each cell line was irradiated at its 50% survival dose: 1.85 Gy for E0771 and 2.49 Gy for 4T07, according to clonogenic survival assays. The cells were irradiated at a dose rate of 1.96±0.54  Gy/min with a standard F1 filter, which corresponds to 2-mm aluminum beam hardening. The voltage was at 320 kVp, and the current was 10 mA. These values also approximate fractionated radiation doses used clinically.

### 2.9 Experimental Spectral Acquisition

The plate spectra were measured as described above for the calibration. Immediately after irradiation, the wells were aspirated and experimental solutions were added. Cells were continuously kept at 37°C during these experiments. Spectra were measured at 0-, 1-, 2-, 4-, 6-, and 24-h post-irradiation. Measurement time points were rounded to the nearest hour, and cells were incubated and stored in a light-free environment between each acquisition.

### 2.10 Microscope Imaging

The BNPs can be visualized in a standard confocal microscope (Zeiss Axio Observer.Z1, Carl Zeiss Microscopy, Cambridge, Massachusetts) with the addition of a Quad-View system (QV2, Photometrics, Tucson, Arizona), which uses a series of beamsplitters and bandpass filters to split light from the sample onto four quadrants of the detection camera (Orca Flash, Hamamatsu, 2048×2048  pixels, Bridgewater, New Jersey). BNP measurements involved a filter set designed to detect light at 470, 500, 535, and 560 nm with a passband of ∼30  nm (Brightline Fluorescence Filters, 560/25  nm, and 500/24  nm, Semrock) and a mercury lamp (X-CITE 120 Lamp, Lumen Dynamics, Mississauga, Ontario). A long-pass fluorescence filter was used to illuminate the sample with excitation light at 430 nm, while the fluorescence and phosphorescence at higher wavelengths were passed through to the detection optics. A background image taken from a cell plate well that contained only the media solution was imaged and was subtracted from the acquired BNP image in MATLAB. The QV2 image was aligned as before by cropping each individual wavelength image and then registering each image with an affine transformation to the 500-nm image. The ratio of the intensity detected from the emission at 565 and 500-nm (F/P) of the BNPs was computed over each image pixel. Because the optics of this system are unlike the plate reader, a microscope-specific calibration curve was obtained in the same manner as described in Sec. 2.4. For each time point (except 24-h post-irradiation), the F/P ratio was microscopically imaged post-plate-reader acquisition. Once the average F/P ratio was obtained over each image, the data were analyzed in the same manner as the spectra.

Immediately after the spectra were obtained with the plate reader, a microscopic image was taken of the same plate before it was placed back in the incubator.

### 2.11 Spectral Analysis and Oxygen Consumption Rate Calculation

As previously described in the BNP calibration, a spectral decomposition in MATLAB was performed for the entire plate. From this, the full fluorescence and phosphorescence spectra were attained, and the F/P ratio was calculated. Then, using the calibration curve, the percent oxygen was quantified for each well. The control groups (BNPs, BNPs with mineral oil, mineral oil alone) were measured: the BNPs reported the expected spectra at 21% $O_{2}$, and the mineral oil did not contribute any significant fluorescence. The BNPs were confirmed to report the expected spectra in a low-oxygen environment via the BNPs + sodium dithionite group, where the sodium dithionite scavenged $O_{2}$ within the well. The remainder of the analysis is in the experimental group: BNPs + cell media. Our first task was to correct for external diffusion [Fig. 3(a)]. The diffusion rate of air into the plate was quantified using the wells without the cells by calculating the rate of change of oxygen. We plotted this diffusion rate against the oxygen values (still using wells without cells) to obtain a curve and fit that accounts for the amount of oxygen in the well via external diffusion. Similarly, the change in oxygen over time was quantified in the group that contained cells; diffusion was corrected for using the equation of the fit described above [Fig. 3(b)]. Finally, the OCR was calculated using the change in oxygen percentage over time, corrected for diffusion effects [Eq. (2)]:

$$\mathrm{OCR}({\%O}_{2},t)=\frac{{\Delta \%O}_{2}}{\Delta t}-D({\%O}_{2}),$$ (2)

where D is diffusion and t is time.

### 2.12 Statistical Analysis

A Bland–Altman analysis was performed to compare the Seahorse and BNP assays. A two-way analysis of variance (ANOVA) was applied to determine if irradiation significantly altered the basal OCR (versus the unirradiated control) followed by Tukey’s post-hoc test. A p<0.05 was considered significant.

## 3 Results

### 3.1 Seahorse Assay and BNP Equivalence

**Fig. 4:**
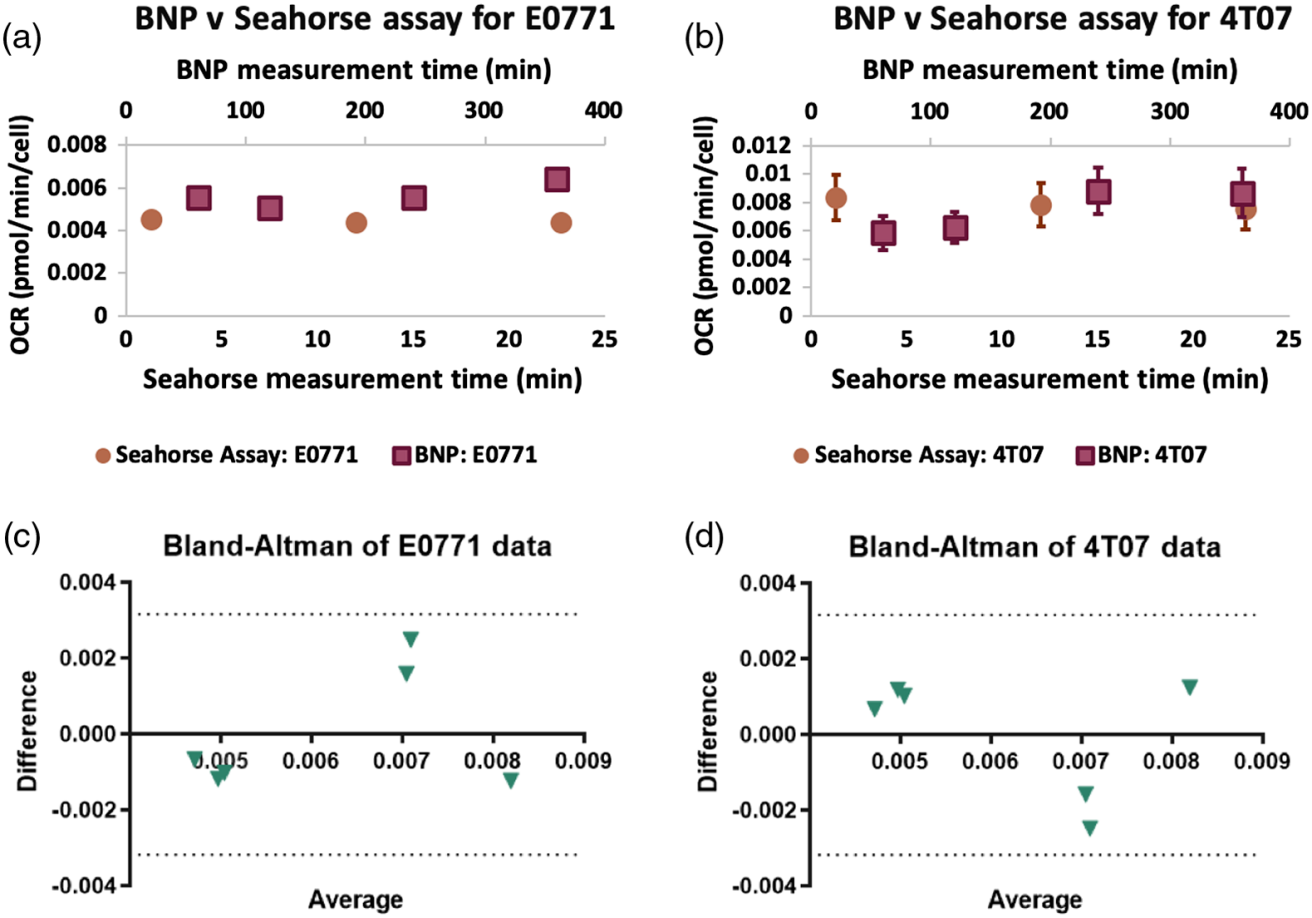
Equivalence of BNP versus Seahorse assay for OCR measurements: For both E0771 (a) and 4T07 (b), we compare the gold-standard Seahorse assay OCR with the BNP OCR measurements. Because of the different measurement time points, 0 to 30 min for the Seahorse assay and 60 to 360 min for the BNPs, there are two horizontal axes. For each cell line, the Seahorse and BNP data are remarkably similar with the mean of six wells approaching the OCR value measured with the Seahorse assay. The error bars are the standard error of the mean, and some error bars are too small to be resolved with the symbol. (c) and (d) The Bland–Altman plots in which the difference between the BNP and Seahorse measurements are plotted against their average. Because the data points are within the dotted 95% confidence interval and are scattered on either side of the x axis, the two methods are considered unbiased and comparative.

Figure 4 compares the Seahorse assay results with the BNP OCR results. Figures 4(a) and 4(b) plot the mean Seahorse data (prior to exposure of any external agents) on the lower x axis, while the mean BNP data are plotted on the upper x axis. The methods appear relatively consistent with each other for both cell lines. Figures 4(c) and 4(d) show the results of a Bland–Altman analysis for each cell line. The difference of the methods versus the average is plotted with the 95% confidence intervals shown in dotted lines. The data points for both cell lines show a scattering above and below the x axis and within the confidence intervals. This shows that the two methods for measuring and quantifying the OCR are equivalent.

### 3.2 Irradiated versus Unirradiated Cells

**Fig. 5:**
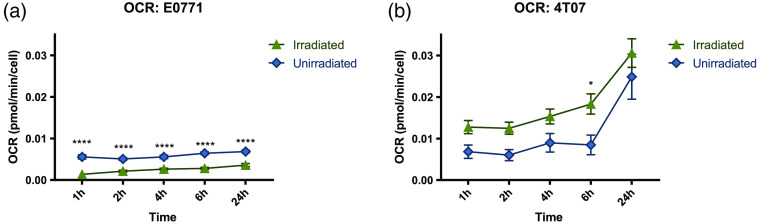
Irradiated and unirradiated OCR for E0771 and 4T07: (a) The irradiated and unirradiated OCR for E0771 and (b) the irradiated and unirradiated OCR for 4T07. Both cell lines were irradiated with their 50% SF, and unirradiated cells were sham irradiated. The error bars represent the standard error of the mean. Notably, both cells lines demonstrate a difference between those exposed and those unexposed to radiation, with E0771 demonstrating a significant difference at all timepoints. Significant p-values of <0.0001 are represented by **** for E0771, and * for 4T07 is p=0.0479 via a two-way ANOVA and Tukey’s multiple comparisons test.

The OCR, with and without irradiation, is shown in Fig. 5 for E0771 and 4T07. The full experimental data can be found in the Supplemental Material (Figs. S8–S10), including the F/P ratios and percent oxygenation. The trends are similar in both cell lines, increasing over time, after receiving their 50% survival fraction (SF) dose. For E0771 [Fig. 5(A)], the unirradiated OCR is consistently larger than the irradiated group. A two-way ANOVA reports significance for each time point (p<0.0001). Conversely, for 4T07 [Fig. 5(B)], the irradiated OCR is larger than the unirradiated and only the first timepoint shows a significant (p=0.0255) difference. Table S2 contains the ANOVA tables in the Supplemental Material.

### 3.3 OCR via Optical Microscopy

**Fig. 6:**
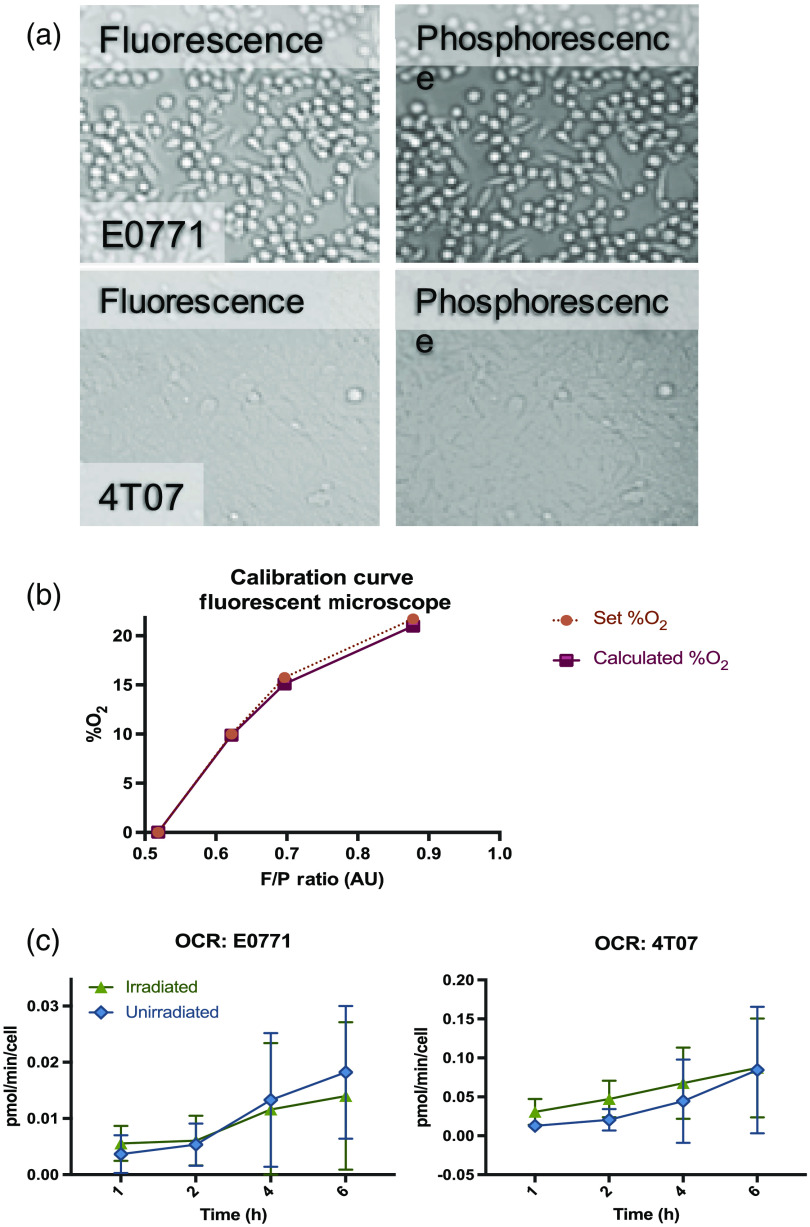
OCR data acquired on microscope system: (a) microscope images of fluorescence and phosphorescence for each cell line using the QuadView filter system. It is worth noting that the microscope does not have a phase-contrast protocol, so the cells are difficult to visualize. (b) The BNP calibration on the microscope shows a quadratic relationship between the F/P ratio and percent $O_{2}$. (c) The OCR of E0771 and 4T07 as calculated from the microscope data is much noisier than the plate reader; however, it should be noted that the analysis averages the entire image not just the area of the cells. The standard deviation bars are quite large, and there is no significance between irradiated versus unirradiated groups. The 4T07 group, compared with the plate reader data, shows the same trend, with the irradiated group having a larger OCR compared with unirradiated.

Figure 6 shows the results of the microscope data. Unlike the plate reader, which is a monochromator, the microscope uses filters that are centered at the fluorescence and phosphorescence emissions. Because of these specific optics and the camera (which may be more sensitive to one end of the spectrum), we calibrated the BNPs in this system by taking images of different percent $O_{2}$ and calibrating it against the F/P ratio as described in Sec. 2.4. In Fig. 6(a), the images show the fluorescence and phosphorescence images for each cell line. Recall that these images are obtained via a QuadView system that splits the image into four quadrants, two of which are centered around 500 (fluorescence) and 564 nm (phosphorescence). The microscope does not have phase-contrast, so the cells are difficult to visualize. Of the easily-visualized cells, the rounded, unhealthy cells (notable in E0771 images) are the most obvious. We did not measure the apoptotic fraction during this experiment, but it is clearly important as fewer cells will lead to a naturally decreased OCR. The calibration [Fig. 6(b)] shows a quadratic relationship between the F/P ratio and the percent oxygen. Figure 6(c) shows the mean OCR results for E0771 and 4T07, comparing irradiated and unirradiated groups. The standard deviation error bars are much larger in the microscope dataset compared with the plate reader. The E0771 does not compare with the plate reader data; conversely, the 4T07 data show an increasing trend with the irradiated cells consuming more oxygen. This is comparable to the plate reader data.

## 4 Discussion

### 4.1 OCRs Quantified via BNP Emissions are a Viable Alternative to the Seahorse Assay

The BNPs show consistency in their response to different oxygen environments over time. While the overall intensity might decrease (though not significantly in our experience after tracking BNPs for six months), the ratiometric intensities remain stable; however, in our experience, the BNPs intensity shows a minor dependence on experimental temperature. For this reason, we maintained 37°C in this experiment (via the plate-reader). The equivalence between the gold-standard Seahorse assay (via the Bland–Altman analysis) with our nanoparticle method highlights the success of this novel ratiometric OCR measurement technique. The main limitation of the Seahorse assay that we sought to overcome is its inability to translate to more complex systems—either 3D cultures or *in vivo*. Our successful OCR quantification of two cell lines opens many possibilities for future studies that require dynamic monitoring of OCR of cells. Furthermore, with modifications to the optical imaging setup in the microscope, imaging during OCR measurements should be possible. By outfitting a mouse with a dorsal window chamber, injecting tumor cells, and applying BNPs, we can measure the ${\mathrm{pO}}_{2}$ across the window using the microscope as described in previous studies.^33^^,^^38^ By measuring the local ${\mathrm{pO}}_{2}$ gradients near vessels and combining these results with theoretical simulations using known oxygen transport parameters, and oxygen diffusivity, we can extrapolate the local OCR across the tumor. This method is similar to the experiment by Dewhirst et al,^46^ in which they used oxygen electrodes and deduced local OCR in rat dorsal window chamber models.

Like other methods for measuring the OCR, oxygen diffusion through permeable materials was significant despite the addition of an oil barrier; however, the substantial effects of diffusion were controlled through the use of control wells (wells without cells). Once we took this diffusion into account, our method via the plate reader produced repeatable, reliable OCRs.

Though the BNPs, when used with a plate reader, are a sensitive and objective method for quantifying OCR, the main advantage is the ability to extend the method to optical imaging. With the addition of a Quad-View filter system and a MATLAB analysis, we can image and quantify fluorescence and phosphorescence (Fig. 6). Not only does that allow *in vitro* analyses of cell heterogeneity and morphology, but it is the only method for quantifying the OCR in tumor spheroids, tumor emboli, and *ex vivo*, live-tissue sections. This is notably different than the Seahorse assay in which specific microplates and cartridges are used to measure the OCR. For heterogenous samples (on slides or in plates) or complex, 3D culture models (that require specialized growing conditions and plates), microscope-based OCR measurements are invaluable. For our system, the microplate is necessary to determine the baseline oxygen percentages due to its straightforward calibration of the BNPs; optical imaging would be a secondary metric for determining relative changes in OCR. The latter is particularly useful because the OCRs may be modulated by extrinsic factors such as environmental hypoxic conditions instead of chemically-induced mitochondrial changes in the cells. Finally, this dual-modality method uses standard technology (plate reader and confocal microscope) found in most labs and; with the exception of the BNPs, there are no expensive cartridges or additives necessary for baseline measurements of OCR.

### 4.2 Noisy Microscope Data Diminishes BNP-Measured OCR Sensitivity

Our data are particularly subject to noise in the microscope with large standard deviations; however, the same trends as shown in the plate reader data are evident (i.e., the irradiated 4T07 cells show a higher OCR than the unirradiated cells). One source of noise that could be more tightly controlled is the depth-of-field; it is well-known that the amount of oxygen in an *in vitro* system is highly dependent on the depth of the measurement. In an unpublished study from our group, the difference between the ${\mathrm{pO}}_{2}$ at the surface level of media was compared with the ${\mathrm{pO}}_{2}$ at the cellular level using an $O_{2}$ microelectrode. For all four cell lines (Hct116, 4T1, 786-0, and R3230 Ac), the ${\mathrm{pO}}_{2}$ at the cellular level was significantly lower (p<10-6, T-test) than at the media surface.^47^ In the microscope, the depth at which we are imaging is variable, depending on where the cells are in focus and including significant background media signal (from intensities at various depths). According to our unpublished study, the ${\mathrm{pO}}_{2}$ will also be inaccurate if we are not imaging at a particular depth. Moreover, the analysis averages the signal over the entire field of view instead of just the cells. A masking protocol for areas of interest (i.e., cells) would likely decrease the uncertainty of the measurements significantly. The plate-reader uses a bottom-read technique that eases this noise effect, providing a more robust OCR measurement. By utilizing a structure illumination technique, the signal recorded at the point of focus (at the cells) would afford a measurement that provides an accurate data point at the cell layer.

### 4.3 OCRs of Irradiated Cell Lines Show Opposite Trends

The OCR measurements across the experimental (irradiated) and baseline (unirradiated) cells were consistent. E0771 demonstrates a highly significant reduction in OCR between irradiated and unirradiated cells. Interestingly, though both cell lines were irradiated at their 50% survival fraction, they demonstrate opposite trends: irradiated E0771 cells exhibit a lower OCR than unirradiated cells, while irradiated 4T07 cells exhibit a higher OCR than unirradiated cells. Processes involved in energy production make up the majority of the oxygen consumption of the basal respiration rate of unirradiated cells, and after irradiation, the observed changes in the OCR of E0771 cells may be indicative of a decrease in the membrane potential required to produce ATP.^22^^,^^48^ The observed increase in OCRs in the 4T07 cells may be attributed to increases in nuclear DNA repair; there are multiple studies that demonstrate these mechanistic correlations in irradiated cancer cells.^48^^–^^50^ For 4T07 specifically, there is likely a correlation between nuclear DNA repair and enhancement of cyclin-dependent kinase 1, pyruvate dehydrogenase kinase 1, and glucose metabolism in response to radiation.^48^^–^^51^ In a study by Qin et al,^48^ three cell lines (MCF10A, HK18, and P + JB6), exhibited a three-fold increase in OCR (measured via a Clarke-type oxygen electrode) over the course of 40 h after a single dose of ionizing radiation. This increase could be the impact of DNA repair, an ATP-consuming process.^48^ While this study, among others,^50^ indicates that the majority of cancerous cell lines demonstrate an increased OCR after radiation exposure, E0771 demonstrates a different trend. For the E0771 cells, we do not see increased OCR post-radiation exposure. E0771 cells have a lower basal OCR and a higher sensitivity to radiation than 4T07, both notable effects of a decreased energy metabolism.^49^ There could be a number of competing effects in these results: depending on the fraction of cells that died via apoptosis and double-strand DNA breaks, the necessity of increasing the ATP consumption to mitigate damage will be smaller. The change from anaerobic metabolism to aerobic metabolism would also modulate oxygen metabolism, depending on factors such as hypoxia-inducible factor 1alpha and other modulators of glucose metabolism. These are only a few of the possibilities that explain the data, but it is clear that a dedicated experiment aimed toward defining the underlying mechanisms for the differential effect of radiation on the OCR of these two cells lines would be required.

## 5 Conclusion

Our data demonstrate a novel method for precisely and accurately quantifying the OCRs of cell *in vitro* via ratiometric oxygen sensing. Our results show comparable values relative to the standard Seahorse assay; however, unlike the Seahorse assay, this method can be extended to *in vivo* studies in which fluorescence imaging is available and optical microscopy imaging. While this method has yet to be tested on human-derived cancer cell lines or in conjunction with chemicals that alter the cellular metabolism, there is a wide field in which BNP-based OCR measurements are effective and advantageous.
