# Supplementary material for: Dual-emissive, oxygen-sensing boron nanoparticles quantify oxygen consumption rate in breast cancer cells
Source: J Biomed Opt. 2020 Nov 23;25(11):116504. doi: 10.1117/1.JBO.25.11.116504 (PMC7682476; doi:10.1117/1.JBO.25.11.116504)
Supplement: Supplementary file 1 [file JBO_025_116504_SD001.pdf]

Supplemental Information

Synthesis and Characterization of Boron Polymer and BNPs

This supplemental section contains supplementary figures describing the preparation and characterization of BNPs.

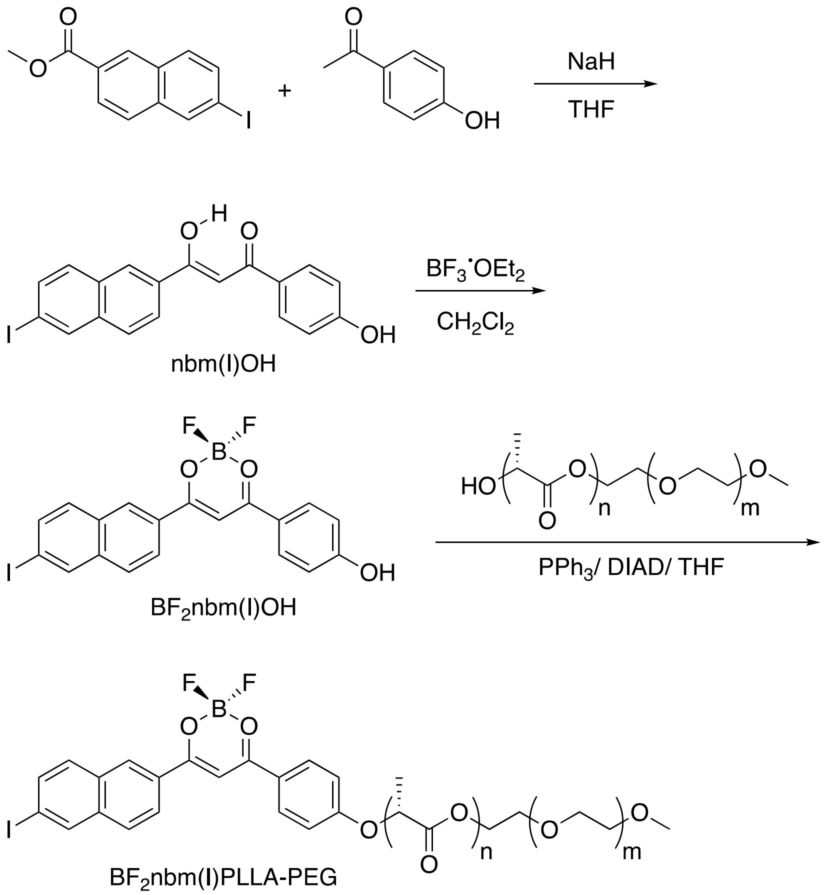

Fig. S1 Schematic of the synthesis of BF2nbm(I)PLLA-PEG.

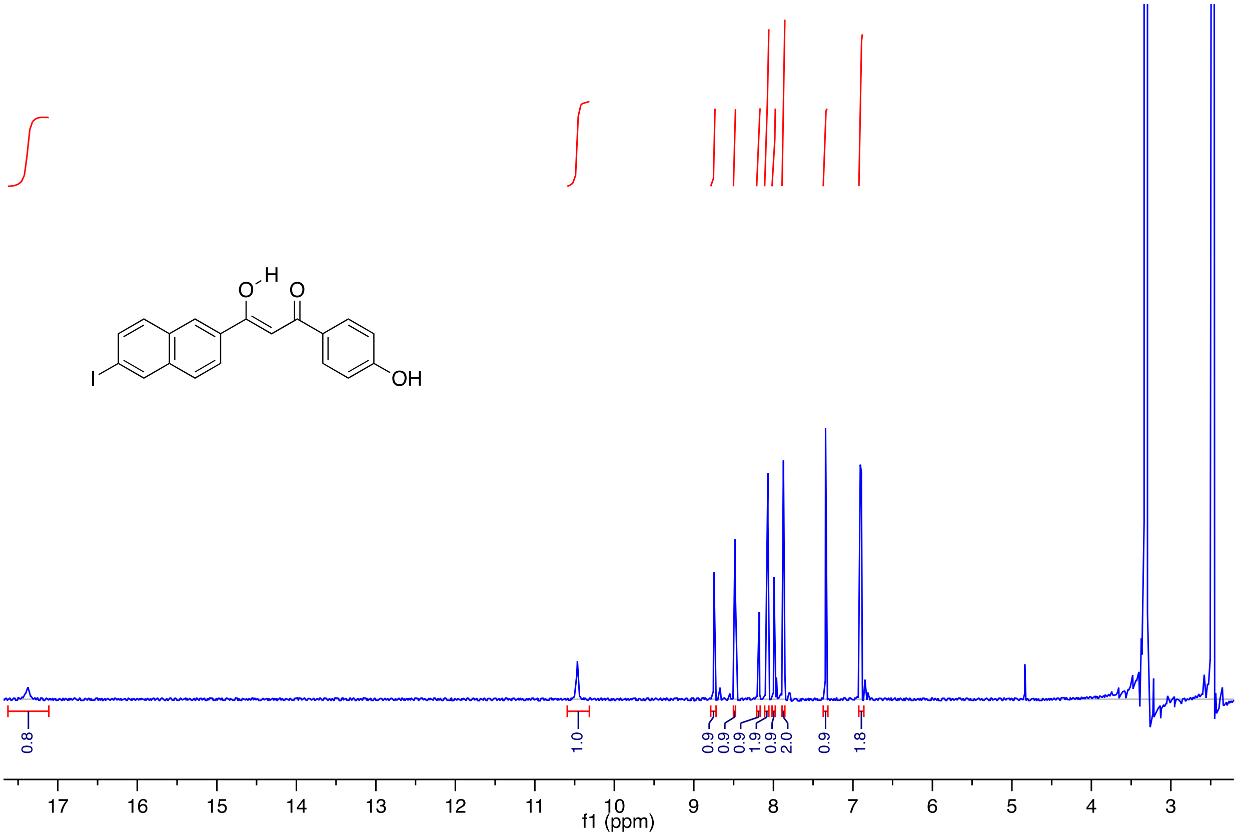

Fig. S2 nbm(I)OH: 1H NMR: (600 MHz, D6-DMSO) δ11.22 (s, broad, 1H, phenol-OH), 8.99 (s, 1H, 1-NpH), 8.55 (s, 1H, 5-NpH), 8.33 (m, 3H, 2, 6-PhH, 4-NpH), (d, J = 6, 1H, 3-NpH), 7.95 (m, 2H, 7, 8-NpH), 7.87 (s, 1H, COCHCO), 7.00 (d, J = 6, 2H, 3, 5-PhH).

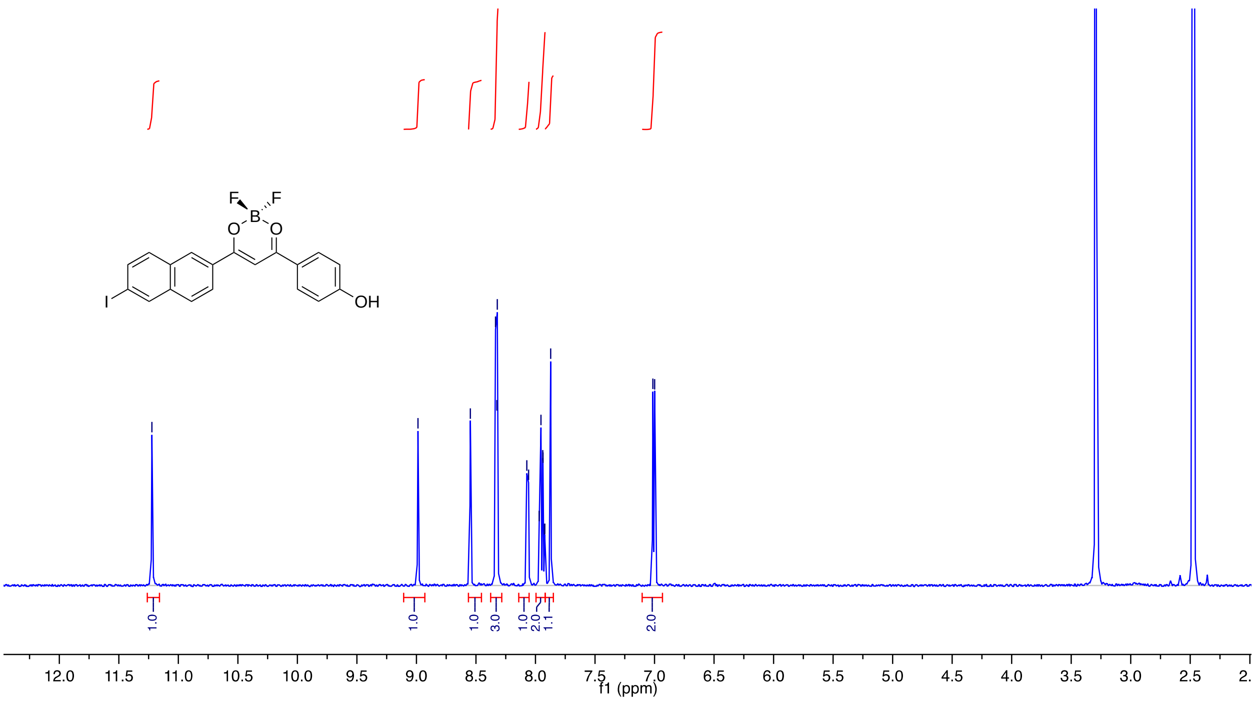

Fig. S3 BF_2nbm(I)OH: ^1H NMR: (600 MHz, D_6-DMSO) δ11.22 (s, broad, 1H, phenol-OH), 8.99 (s, 1H, 1-NpH), 8.55 (s, 1H, 5-NpH), 8.33 (m, 3H, 2, 6-PhH, 3-NpH), 8.07 (d, J = 6, 1H, 4-NpH), 7.95 (m, 2H, 7, 8-NpH), 7.87 (s, 1H, COCHCO), 7.00 (d, J = 6, 2H, 3, 5-PhH).

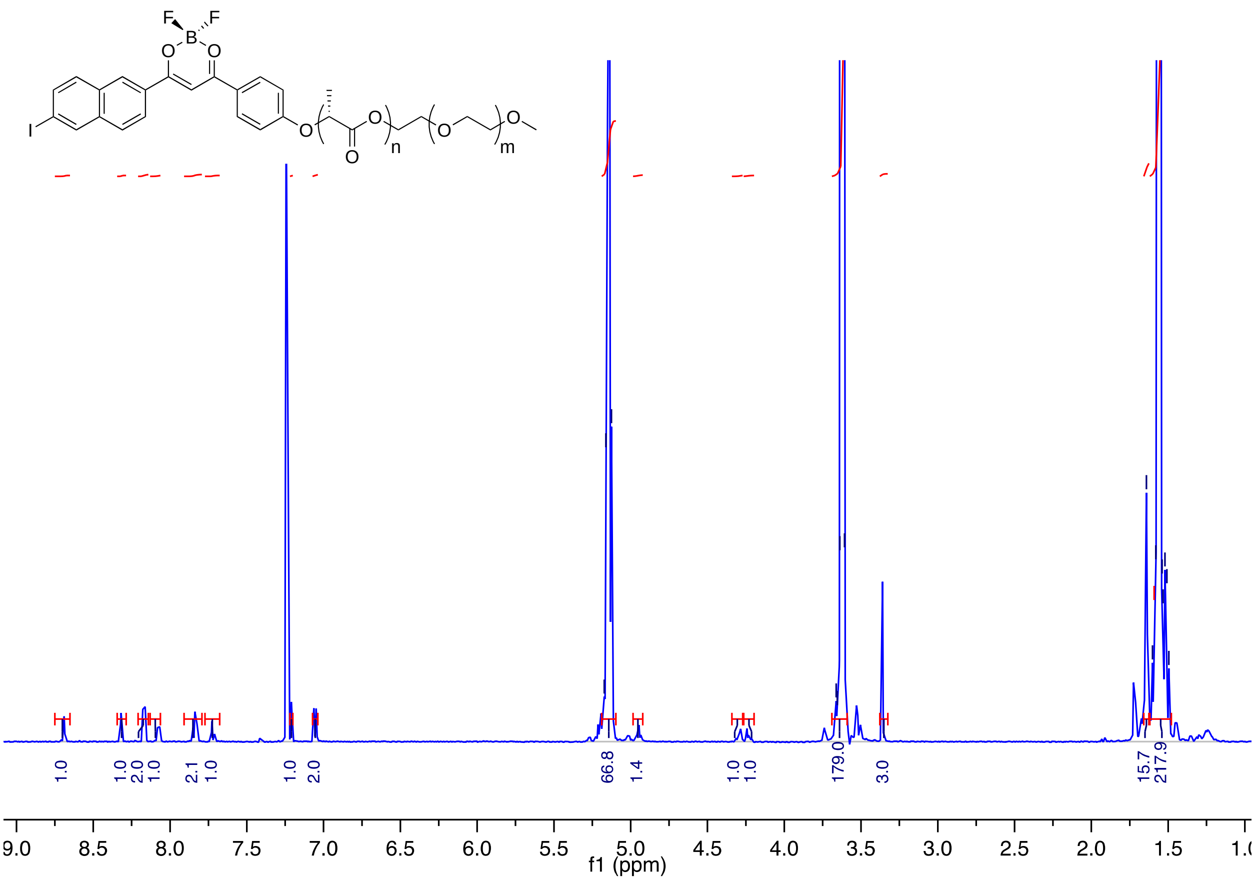

Fig. S4 BF2nbm(I)PLLA-PEG. 1H NMR: (600 MHz, CDCl3) δ8.69 (s, 1H, 1-NpH), 8.32 (s, 1H, 5-NpH), 8.17 (d, J = 6, 2H, 2, 6-PhH), 8.09 (d, J =12, 1H, 3-NpH), 7.85 (m, 2H, 7, 8-NpH), 7.72 (d, J = 12, 1H, 4-NpH), 7.21 (s, 1H, COCHCO), 7.06 (d, J = 6, 2H, 3, 5-PhH), 5.17 (q, J = 6, 66H, PLLA-H), 3.62 (s, broad, 179H, PEG-OCH2CH2-), 3.36 (s, 3H, PEG-OCH3), 1.55 (m, broad, 217H, PLLA-CH3).

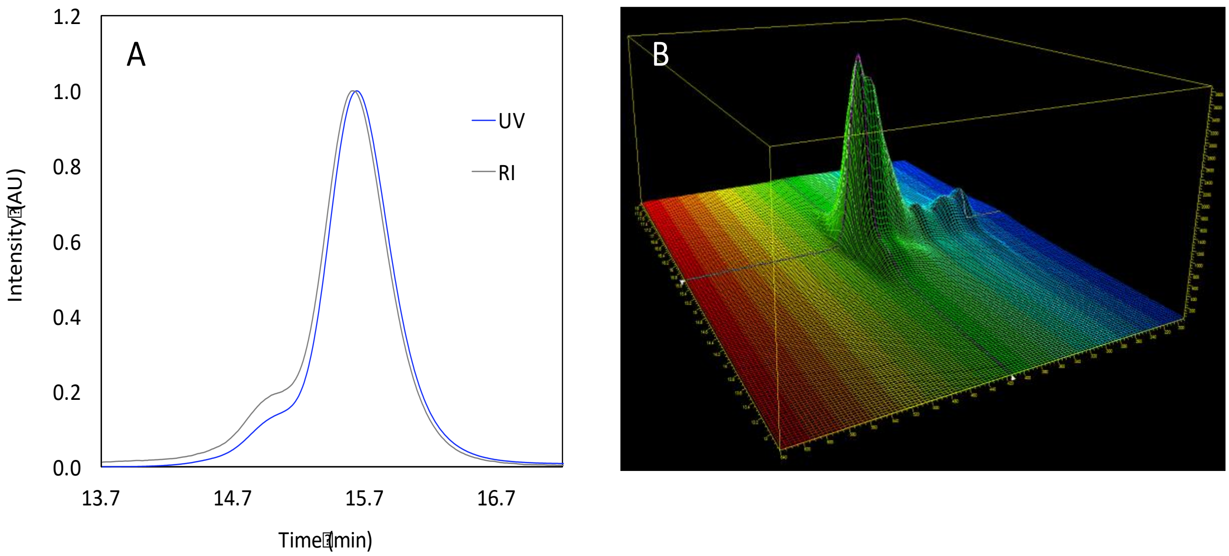

Fig. S5 GPC Traces of BF2nbm(I)PLLA-PEG.

(A) 2D plot of intensity vs elution time (UV = absorbance and RI = refractive index signal). **(B)** 3D chromatogram of the polymer (Y axis = intensity, X axis = absorbance wavelength, Z axis = elution time).

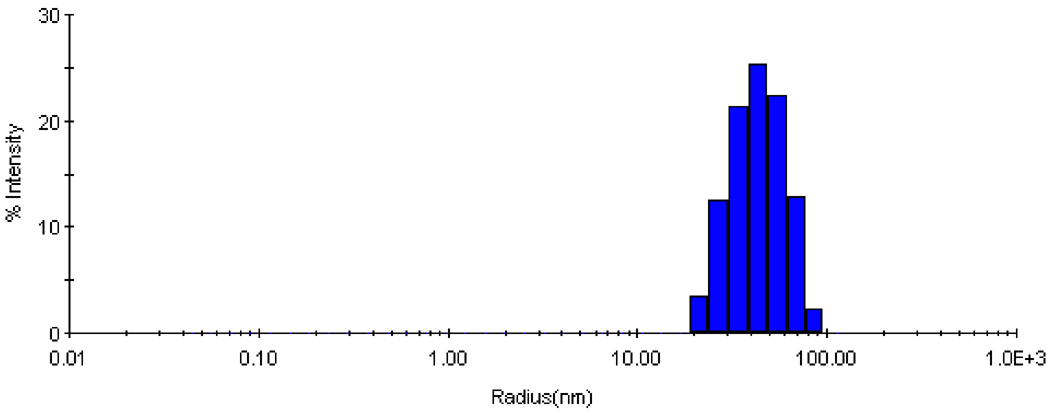

Fig. S6 Dynamic light scattering (DLS) measurement of boron nanoparticles (R_H = 41.5 nm, %Pd = 17.1)

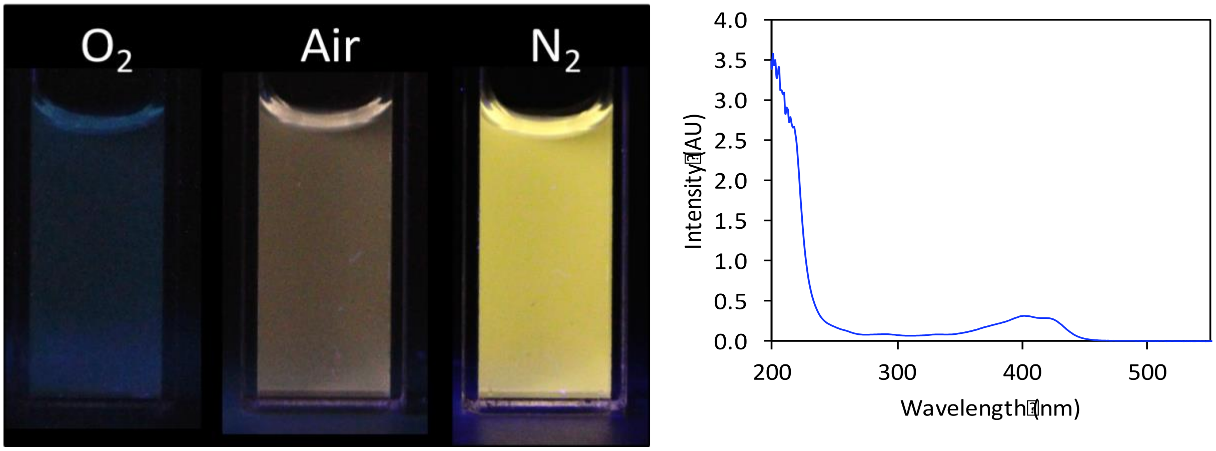

Fig. S7 Left: Images of boron nanoparticles under various conditions with UV excitation (λ_{ex} = 369 nm). **Right:** UV/Vis spectrum of boron nanoparticles in aqueous suspension.

Experimental Data

This section contains supplementary figures relating to the *in vitro* experiment including the plate layout and data from each of the three repetitions of the experiment.

Table S1 96-well plate layout for experiment**.**

	1	2	3	4	5	6	7	8	9	10	11	12
A	BNP+DI (no oil)	BNP+DI (no oil)	BNP+DI (no oil)	BNP+DI (no oil)	BNP+DI +Oil	BNP+DI +Oil	BNP+DI +Oil	BNP+DI +Oil	Oil	Oil	Oil	Oil
B	BNP+ media	BNP+ media	BNP+ media	BNP+ media	BNP+ media	BNP+ media	BNP+ media	BNP+ media	BNP+ media	BNP+ media	BNP+ media	BNP+ media
C	BNP+ media	BNP+ media	BNP+ media	BNP+ media	BNP+ media	BNP+ media	BNP+ media	BNP+ media	BNP+ media	BNP+ media	BNP+ media	BNP+ media
D	BNP+ Sodium Dithionite	BNP+ Sodium Dithionite	BNP+ Sodium Dithionite	BNP+ Sodium Dithionite	BNP+ Sodium Dithionite	BNP+ Sodium Dithionite	BNP+ Sodium Dithionite	BNP+ Sodium Dithionite	BNP+ Sodium Dithionite	BNP+ Sodium Dithionite	BNP+ Sodium Dithionite	BNP+ Sodium Dithionite
E												
F												
G												
	Description of wells by Row				**Row A** contains control wells for BNP and oil		**Row B** contains the experimental wells with either E0771 or 4T07 cells		**Row C** contains control wells of BNP+media without cells		**Row D** contains positive controls: 6 Wells W/Cells 6 Wells W/O Cells	

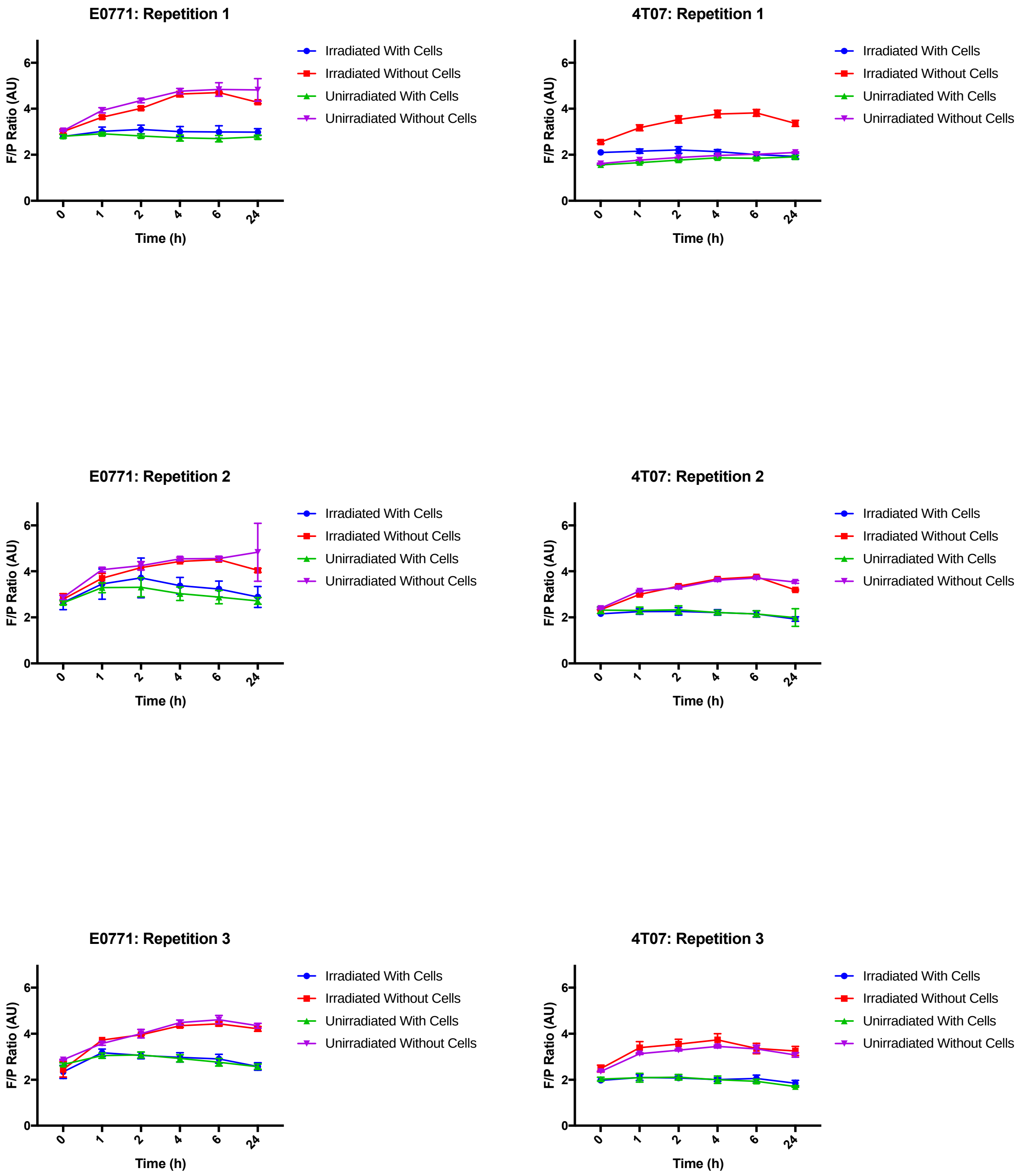

Fig. S8 The mean F/P ratio for each of the experimental repetitions as well as their controls (no cells). Error bars represent the standard deviation.

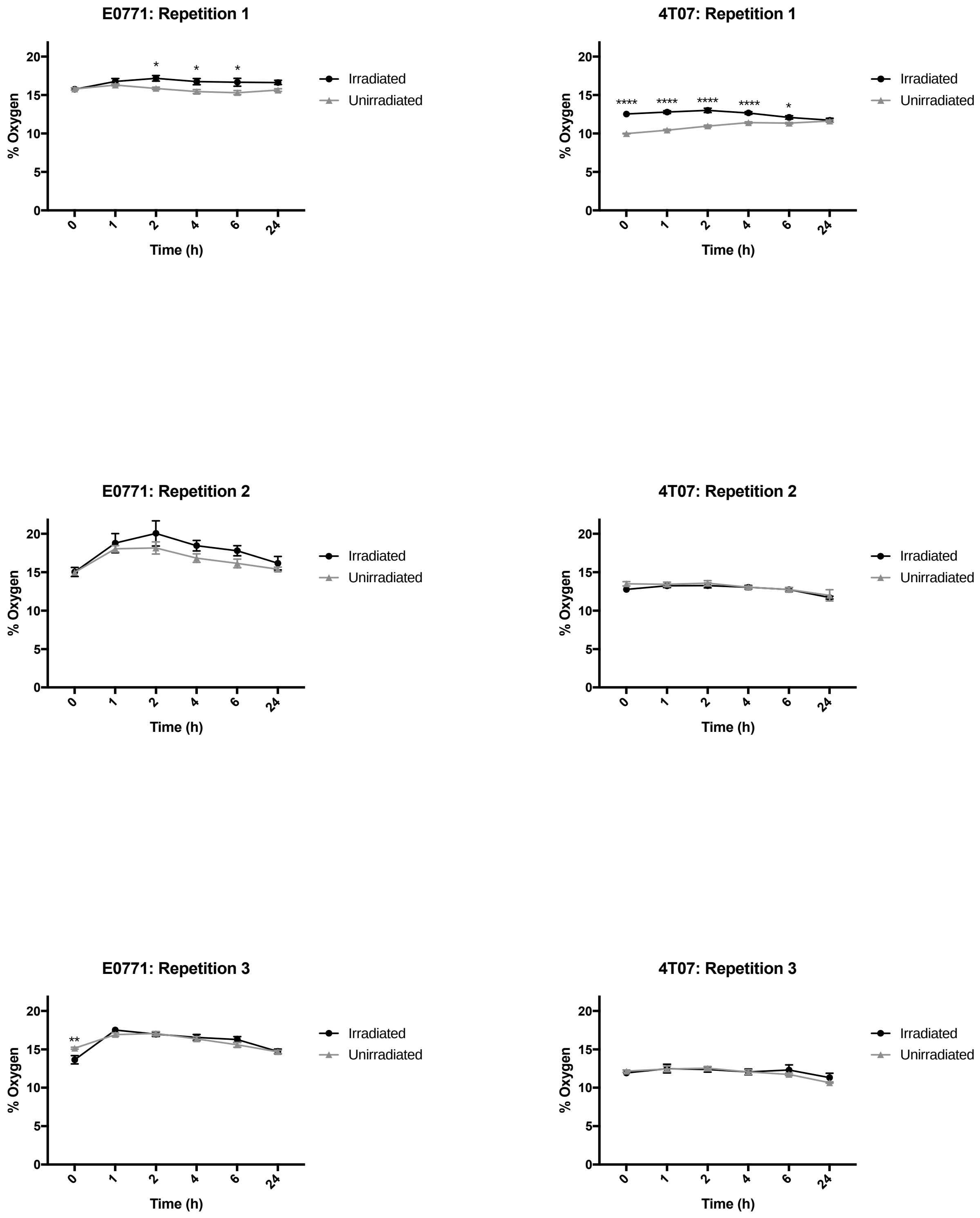

Fig. S9 The percent oxygen for each repetition and cell line, calculated from the calibration curve between the F/P ratio and the percent O_2. The error bars represent the standard error of the mean. Significant p-values are noted, where * corresponds to $p<0.05$, ** corresponds to $p<0.01$, and **** corresponds to $p<0.0001$.

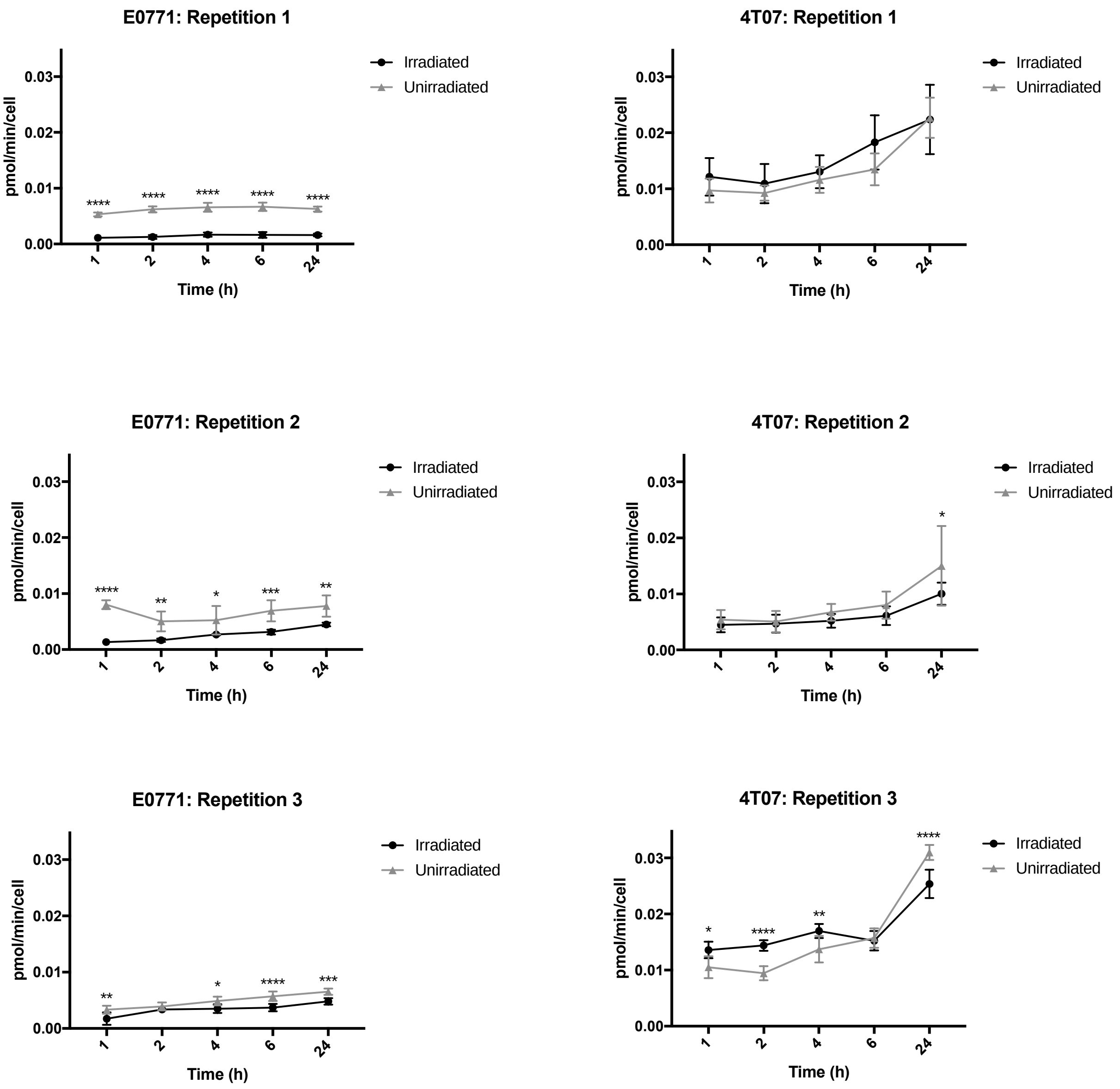

Fig. S10 The OCR for each repetition and cell line. The error bars represent the standard error of the mean. Significant p-values are noted, where * corresponds to p<0.05, ** corresponds to p<0.01, and **** corresponds to p<0.0001.

Table S2 Two-Way ANOVA Tables for both 4T07 and E0771

ANOVA table: OCR E0771	SS (Type III)	DF	MS	F (DFn, DFd)	P value
Interaction	9.734e-006	4	2.433e-006	F (4, 163) = 1.292	P=0.2754
Time	7.473e-005	4	1.868e-005	F (4, 163) = 9.918	P<0.0001
Irradiated v Unirradiated	0.0004985	1	0.0004985	F (1, 163) = 264.6	P<0.0001
Residual	0.0003071	163	1.884e-006		

ANOVA table: OCR 4T07	SS (Type III)	DF	MS	F (DFn, DFd)	P value
Interaction	1.035e-004	4	2.588e-005	F (4, 165) = 0.2031	P=0.9364
Time	0.008177	4	0.002044	F (4, 165) = 16.04	P<0.0001
Irradiated v Unirradiated	0.002041	1	0.002041	F (1, 165) = 16.02	P<0.0001
Residual	0.02103	165	1.274e-004		
